# Cervicovaginal Microbiome Factors in Clearance of Human Papillomavirus Infection

**DOI:** 10.3389/fonc.2021.722639

**Published:** 2021-07-28

**Authors:** Wenkui Dai, Hui Du, Shuaicheng Li, Ruifang Wu

**Affiliations:** ^1^Department of Obstetrics and Gynecology, Peking University Shenzhen Hospital, Shenzhen, China; ^2^Institute of Obstetrics and Gynecology, Shenzhen Peking University-The Hong Kong University of Science and Technology (PKU-HKUST) Medical Center, Shenzhen, China; ^3^Shenzhen Key Laboratory on Technology for Early Diagnosis of Major Gynecologic Diseases, Shenzhen, China; ^4^Department of Biomedical Engineering, City University of Hong Kong, Hong Kong, China

**Keywords:** cervical cancer, high-risk HPV, CVM-targeted intervention, CVM-derived product, HPV clearance

## Abstract

Persistent high-risk human papillomavirus (hrHPV) infection is the highest risk to cervical cancer which is the fourth most common cancer in women worldwide. A growing body of literatures demonstrate the role of cervicovaginal microbiome (CVM) in hrHPV susceptibility and clearance, suggesting the promise of CVM-targeted interventions in protecting against or eliminating HPV infection. Nevertheless, the CVM-HPV-host interactions are largely unknown. In this review, we summarize imbalanced CVM in HPV-positive women, with or without cervical diseases, and the progress of exploring CVM resources in HPV clearance. In addition, microbe- and host-microbe interactions in HPV infection and elimination are reviewed to understand the role of CVM in remission of HPV infection. Lastly, the feasibility of CVM-modulated and -derived products in promoting HPV clearance is discussed. Information in this article will provide valuable reference for researchers interested in cervical cancer prevention and therapy.

## Introduction

Persistent high-risk human papillomavirus (hrHPV) infection is the highest risk to invasive cervical cancer (ICC), which has caused an estimated 570,000 new cases and 311,000 deaths in 2018 (1). Prophylactic vaccines are effective in preventing HPV infection, but providing limited protection against pre-existing HPV infection which impact large populations in developing countries for a long-lasting period (2, 3). It will be an imperative alternative to prevent HPV-infected cervical intraepithelial neoplasia (CIN) and ICC by eliminating HPV infection. An increasing number of literatures suggests the association of natural HPV clearance and CIN regression with cervicovaginal microbiome (CVM) (4–8), which modulate a finely-tuned immune responses balancing reproductive tolerance with protection against genital infections (9). Our and other studies demonstrated predominance of one or few *Lactobacillus* species in CVM of healthy lower reproductive tract (LRT), including *Lactobacillus crispatus* (community-state type I, CST I), *Lactobacillus gasseri* (CST II), *Lactobacillus iners* (CST III) and *Lactobacillus jensenii* (CST V) (10–14). These *Lactobacillus* species benefit reproductive health by inhibiting pathogens *via* produced bacteriocins, lactic acid and hydrogen peroxide (15).

Emerging reports demonstrate imbalanced CVM in women with HPV infection, including increased bacterial diversity, depletion of *Lactobacillus* as well as identified high rate of natural HPV clearance in women with predominant *L. crispatus* in CVM (4, 8, 16–21). To the best of our knowledge, there is no public report investigating the mechanism of interaction between HPV and microbiome, due to difficulties to cultivate HPV *in vitro* and limited mouse models for HPV-medicated cervical dysplasia or cancer. Nevertheless, a number of studies support the concept that CVM modulates immune microenvironment through microbe- or microbe-host interactions to impact the risk of viral infections and clearance (9, 22–25). For instance, *Lactobacillus* conferred colonization resistance to *Gardnerella vaginalis* which induced suppressive immune responses beneficial to persistent HPV infection (22). M N Anahtar et al. demonstrated that CVM was the main modulator of immune responses in lower reproductive tract (LRT) and affected the risk of human immunodeficiency virus (HIV) infection (23). Peptidoglycans (PGN) produced by isolated vaginal *L. crispatus* activate Langerhans cells (LCs), which is the most important antigen presenting cells (APCs) in cervical epithelium (25), and several follow-up investigations further suggest a strong *in vivo* relationship between LCs activities and HPV clearance (26–28).

In this review, we first summarize the association of CVM with HPV infection and clearance, then discuss mechanisms of microbiome, host responses and HPV interaction. Lastly, several potentials are explored about how to eliminate pre-existing HPV infection *via* microbiome-derived products or microbiome-targeted interventions.

## Imbalanced CVM in HPV Infection

**Figure 1:**
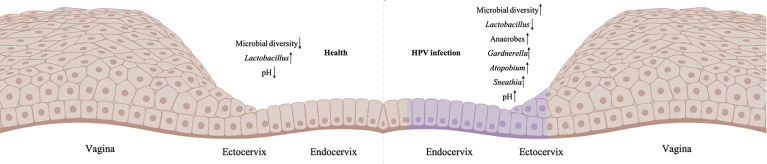
Imbalanced CVM in HPV-infected cervix. The left is the normal cervicovaginal microenvironment without HPV infection, and the right is HPV-positive microenvironment (HPV-infected cells are labelled purple). Created in BioRender.com.

Emerging evidence suggests association between CVM and HPV infection and persistence. Almost all cross-sectional studies consistently found higher diversity of CVM in HPV-positive women, with or without CIN, as compared to HPV-negative individuals (16–21, 29–32). In recent decade, a growing body of literature suggests that depletion of *Lactobacillus* and overgrowth of anaerobic bacteria is associated with increased CVM diversity (**Figure 1**) (16–21, 29, 30). For individuals infected with HPV but without CIN or ICC, initial cross-sectional studies involving Korean (n=68 selected from 912 women in Healthy Twin Study) and Chinese (n=70) women identified reduced levels of *Lactobacillus* as well as higher abundance of bacterial vaginitis (BV)-associated bacteria such as *Gardnerella*, *Sneathia* and *Megasphaera* (18, 30). This is consistent with increased susceptibility to HPV infection in women with BV revealed by meta-analysis (33). Besides to *Gardnerella*, *Sneathia* and *Megasphaera*, additional reports found greater relative abundance of *Atopobium*, *Bacteroides*, *Prevotella* and lower proportion of *Lactobacillus* in CVM of HPV-positive women (16, 17, 29). Studies involving women with CIN or ICC consistently found significant decrease of *Lactobacillus* and substantial increase in CVM diversity compared with HPV-negative individuals (18, 19, 31, 32).

At species level of *Lactobacillus*, a marked decrease of *L. crispatus* was found in CVM of women with HPV infection, CIN or ICC, while *L. iners*-dominant CVM had higher risk of CIN (8, 18, 31, 32, 34). Additionally, women with HPV infection had accumulation of *Bacteroides plebeius*, *Acinetobacter lwoffii*, *Prevotella buccae*, *Dialister invisus*, *G. vaginalis*, *Prevotella buccalis* and *Prevotella timonensis* in CVM (29, 31, 32, 34). For instance, a study involving 70 women with CIN and 50 HPV-negative women indicated that 6-fold risk of CIN associated with unique CVM, which is characterized by paucity of *L. crispatus*, enriched *A. vaginae*, *G. vaginalis* and *L. iners* (30). Two independent systematic reviews and meta-analysis also found that *L. crispatus* correlated with decreased risk of hrHPV infection and CIN (35, 36). Compared with *L. crispatus*-dominant CVM, women with non-*Lactobacillus*- or *L. iners*-dominant CVM had 2-3 times higher odds of hrHPV prevalence and CIN, as well as 3-5 times higher odds of any prevalent HPV (95% CI) (35).

Besides to microbial components, emerging literature explores functional difference of CVM between HPV-positive and HPV-negative women (37–39). Functional prediction of 16S rDNA amplicon sequencing data found accumulation of multiple pathways in HPV-infected and CIN women, including those of folate biosynthesis and oxidative phosphorylation (37). Metagenomic analysis of 17 CIN, 12 ICC cases and 18 healthy individuals found enriched genes related to peptidoglycan synthesis as well as depletion of dioxin degradation and 4-oxalocrotonate tautomerase in CVM of women with CIN or ICC (38). Biofilm formation assessment identified higher formation rate in HPV-positive women (45%) compared to HPV-negative women (21.9%) (39), which may be attributed to increased levels of obligate anaerobic bacteria in CVM of HPV-infected women, such as *G. vaginalis* with sialidase-encoding gene involved in biofilm formation (8).

Above-mentioned observational studies are only possible to demonstrate association of CVM with HPV infection and CIN diseases rather than causality. Longitudinal data is increasingly applied to explore the causal link (7, 40, 41), which has profound clinical impact to provide effective alternatives for therapeutic strategies of HPV-infected CIN. Six-month follow-up of 211 Nigerian women showed the association of *Lactobacillus* paucity and high CVM diversity with persistent hrHPV infection (40). Analysis of serial cervicovaginal specimens obtained over 8-10 years unraveled that high relative abundance of *L. crispatus* in CVM had the lowest risk of HPV infection compared to other types of CVM, according to 16S V1-V2 rRNA gene amplicon sequencing and HPV DNA testing conducted annually (41). Brotman and colleagues collected self-sampled mid-vaginal swabs twice a week for 16 weeks from 32 reproductive-age women, and showed that depletion of *Lactobacillus* in CVM may increase the chance to acquire transient and persistent HPV infection (7). Consistently, meta-analysis involving 39 articles suggests the protection against HPV infection imposed by *Lactobacillus*-dominant CVM (42). Another systematic review and meta-analysis of longitudinal studies also support a causal relationship between non-*Lactobacillus*-dominant CVM and cervical carcinogenesis *via* the effect of CVM on HPV infection (RR 1.33, 95% CI) and persistence (RR 1.14) (43).

## CVM is Associated With Natural HPV Clearance and CIN Regression

**Figure 2:**
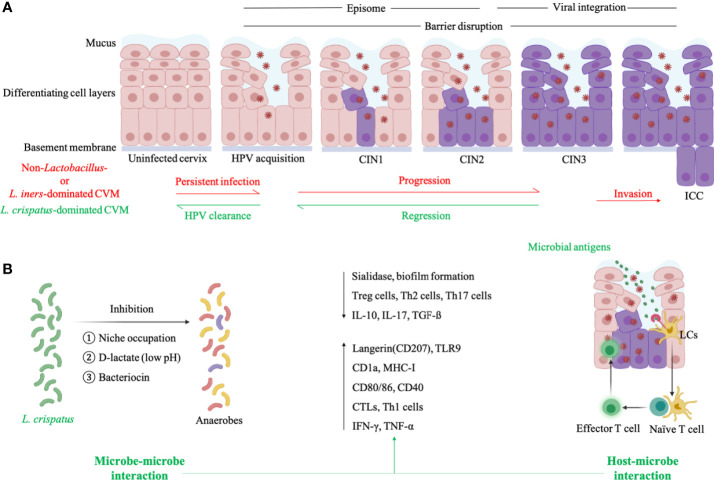
Cervicovaginal microenvironment in persistent HPV infection and natural HPV clearance. **(A)** *L. iners*- or non-*Lactobacillus*-dominated CVM is characterized by allowing the proliferation of anaerobes which produce sialidase to disrupt epithelial barrier and then facilitate the entry of HPV. HPV particles exist in infected keratinocytes as episomes before entry into the nucleus, and viral integration induces high expression of E6/E7 proteins to promote abnormal cell proliferation as well as carcinogenesis. *L. crispatus*-dominated CVM is associated with natural HPV clearance and CIN regression. **(B)** For HPV-positive women with *L. crispatus*-dominated CVM, *L. crispatus* can inhibit growth of anaerobes through several mechanisms (microbe-microbe interaction) and activate LCs which is the only APCs in cervical epithelium to present HPV antigens and induce HPV-specific CMI (host-microbe interaction). Both microbe- and host-microbe interaction decrease the level of factors correlated with barrier impairment (sialidase and biofilm of anaerobes) and suppressive immunity (Treg, Th2, Th17 cells, IL-10, IL-17, TGF-ß), and increase the expression of biomarkers in activated LCs (antigen-binding langerin and TLR9, antigen-presenting CD1a and MHC-I, co-stimulatory molecule CD80/86 and CD40) as well as CMI-associated molecules (cytotoxic T lymphocytes-CTLs, Th1 cells, IFN-γ, TNF-α). This figure applied icons in BioRender.com.

According to a follow-up analysis on 55 women with HPV infection and 17 age-matched healthy HPV-negative women, *L. crispatus* was the most abundant *Lactobacillus* species in individuals with natural HPV clearance (**Figure 2A**) (8). Conversely, high proportion of *Atopobium* in CVM had significantly slowed HPV remission rate in 16-week follow-up, compared to *L. crispatus*-dominant CVM^8^. Another longitudinal study involving 64 HPV16-positive women found more frequent transition between identified CSTs, including dominant *Lactobacillus* sp., *L. iners*, two mixed non-*Lactobacillus* of CVM, in women with persistent HPV16 infection (34% with averaged 155.5 days interval) when compared to women with natural clearance of HPV16 (19% with averaged 162 days interval) (**Figure 2A**) (6). Consistently, Anita Mitra and partners found more stable CVM in women with CIN2 regression, as compared to individuals with CIN persistence or progression^4^. In this study, 87 CIN2 patients aged 16-26 years old were included in two-year follow-up showing that women with *Lactobacillus*-dominant CVM at baseline are more likely to regress at 12 months while slower regression was associated with *Lactobacillus* depletion as well as increased abundance of *Megasphaera*, *Prevotella timonensis* and *G. vaginalis* (4). At species level, women with *L. crispatus*-dominant CVM had faster regression and higher rate of CIN remission at 12 and 24 months (4).

A total of four CSTs, dominated by *L. crispatus*, *L. iners*, *G. vaginalis* and mixed genus, was identified in another study involving 273 women aged 18-25 years old (5). At first visit, *Lactobacillus* and *Gardnerella* abundance was associated with CIN2 regression and progression respectively. Second visit was conducted at least 305 days after first visit, and CIN2 progression had strong correlation with increased bacterial diversity. Functional prediction of 16S rDNA amplicon sequencing data further showed the positive relationship between pathway of cell motility and CIN2 regression, while progression was in association with “Xenobiotics Biodegradation and Metabolism” pathway in CVM.

Fungal components in CVM were also associated with HPV-infected CIN regression (5). Mykhaylo Usyk and colleagues found the protective effect of fungal diversity against CIN progression (OR=0.90, 0.82-1.00) (5). Among fungus *Candida*, *Malassezia* and Sporidiobolaceae, the accumulation of *Candida* was identified in CVM of CIN1 which had the highest regression rate (5). Additionally, a retrospective investigation on 100,605 women who had 2 smears each over a period of 12 years, found that common fungus *Candida* in cervicovaginal microenvironment decreased the risk of squamous intraepithelial lesions (44).

## Microbe- and Host-Microbe Interactions in HPV Clearance

The complexity of cervicovaginal microenvironment of women with HPV infection is determined by HPV, CVM and the host. To explore the contribution of CVM to promote or protect against HPV infection, there is much work to be done in exploring microbe-microbe interactions in CVM, as well as the interactions between microbe and HPV/host (**Figure 2B**).

Sialidase are a group of mucin-degrading enzymes produced by BV-associated *G. vaginalis* and *Prevotella*, and disrupt the integrity of mucosa as well as epithelium to aid the entry of HPV to basal keratinocytes (**Figure 2A**) (45). Besides to compromised cervical epithelial barrier, BV-associated anaerobes also impact several cellular pathways to enable persistent viral infection and subsequent disease development (46–50). *Sneathia* spp., commonly accumulated in CVM of BV and HPV-infected patients, belongs to *Fusobacterium* genus which can activate proinflammatory pathways and inhibit immunocytotoxicity to promote carcinogenesis (51). This information may explain the high susceptibility to HPV infection in women with BV and accumulation of vaginal obligate anaerobic bacteria in women with persistent HPV infection or cervical dysplasia progression (16–18, 29, 30, 33).

Vaginal *Lactobacillus* spp. can produce a large amount of lactic acid through glycogen fermentation, maintaining acidic environment to inhibit the colonization of several pathogenic species such as *Chlamydia trachomatis*, *Neisseria gonorrhoeae* and BV-associated *G. vaginalis* (**Figure 2B**) (15, 52–55). Bacteriocins produced by vaginal *Lactobacillus* also exhibit inhibitory effects on common pathogenic bacteria and certain fungi, such as *G. vaginalis* and *Candida albicans* (**Figure 2B**) (15, 56, 57). In addition, *Lactobacillus* hold the potential to alter surface tension and thus bacterial adhesion which is pivotal in biofilm formation *via* excreted biosurfactants, therefore preventing overgrowth of pathogenic anaerobes, especially *G. vaginali*s (**Figure 2B**) (22, 58–60). Another defense factor derived from vaginal *Lactobacillus* is H_2_O_2_, which destroys vaginal bacterial components with limited expression of H_2_O_2_-degrading enzymes, including *Prevotella* and *Gardnerella* (60, 61). Besides direct inhibition on pathogens, *Lactobacillus* can occupy possible niches to indirectly protect against pathogen colonization (**Figure 2B**). For instance, epithelium adhesin facilitates the adhesion of *L. crispatus* to genital mucosa and then additionally inhibits pilus-mediated adhesion of *G. vaginalis* (22).

As discussed above, vaginal *Lactobacillus* play critical roles in cervicovaginal health, but not all *Lactobacillus*-dominant CVM benefit the host in the same manner. Lactic acid has D- and L-isomer while the former is mainly produced by *L. jensenii*, *L. crispatus*, *L. gasseri* and the latter is produced by *L. iners* and a variety of anaerobes (62). Women with *L. iners*- or non-*Lactobacillus*-dominant CVM therefore have a higher ratio of L- and D-lactate, increasing the expression of extracellular matrix metalloproteinase inducer and activating matrix metalloproteinase 8, which facilitate the entry of HPV to the basal keratinocytes by altering cervical integrity (**Figure 2A**) (62). Conversely, *L. crispatus*-dominant CVM can lead to increased cervicovaginal mucus viscosity and promote viral capture (63). Additionally, CVM predominated by *L. iners* is more instable than CVM with other dominant *Lactobacillus* species and therefore allows growth of strict anaerobes resulting in transition to non-*Lactobacillus*-dominant CVM (4, 64). This is consistent with findings that *L. iners*-dominant CVM tends to be identified in women with persistent HPV infection and progression of cervical diseases (**Figure 2A**) (8, 18, 31, 32, 34). On the contrary, *L. crispatus*-dominant CVM has the lowest possibility in transition to other CVM types (4, 13, 64), and is thus positively associated with cervicovaginal health (**Figure 2A**).

Though many clues exist in microbe-microbe interactions, there are no published reports exploring the mechanism of interaction between CVM and HPV, due to the difficulties of *in vitro* HPV cultivation. Nevertheless, a growing number of literatures demonstrate unique host immune responses (**Figure 2B**) (65), which mediate the CVM-HPV interactions in women with HPV infection. Oncoproteins of hrHPV can suppress presentation of hrHPV antigens and impair alarm functions of infected basal keratinocytes where HPV thrive. For example, hrHPV E7 protein can lead to repression of major histocompatibility complex I (MHC I), LMP2 as well as TAP1 gene through interaction with MHC I promoter, and E5 protein blocks the transport of MHC I and CD1d to the cell surface, which is crucial for HPV antigen presentation (66–71). Infection of hrHPV also reduce the expression of infected keratinocyte-derived chemokine (C-C motif) ligand 20 (CCL20) (72, 73), attracting the migration of LCs which is the only APCs in vaginal epithelium where HPV infection occurs. In addition, hrHPV infection is associated with suppressed LCs activities, including decreased levels of E-cadherin remaining LCs in infected epidermis to capture HPV antigens, and antigen-binding langerin as well as TLR (74–78). Once internalizating HPV antigens, LCs become mature and migrate to lymph nodes *via* chemokine (C-C-motif) receptor 7 (CCR7) on cell surface. However, prior reports found reduced expression of CCR7 in ICC patients, and identified decreased levels of LCs-membrane antigen-presenting and co-stimulatory molecules in exposure to HPV virus-like particles (VLPs), including CD1a, MHC I, CD40 and CD80/86 (66, 67, 75, 76, 79–81).

To the best of our knowledge, no report established *in vivo* CVM-LCs relationship, which can partly improve the understanding of CVM-HPV interactions. Nevertheless, *in vitro* experiment found that a *L. crispatus* strain isolated from vagina activated LCs *via* cell wall-derived PGN, being assessed by elevated expression of TLR (**Figure 2B**) (25). This is consistent with prior findings that TLR agonists promote LCs activation and the induction of HPV-specific cell-mediated immunity (CMI) (77, 78, 82). Candin, produced by *Candida* which is inversely associated with HPV infection, can induce proliferation of T cells to enhance the effect of therapeutic vaccines against HPV (44, 83, 84). Herbst-Kralovetz MM and colleagues also found significant differences of CVM and cervical immune microenvironment between HPV-negative women (n=18), HPV-infected individuals without squamous intraepithelial lesion (n=11), HPV-positive women with low (n=12)/high (n=27) intraepithelial lesion, and ICC patients (n=10) (85–88). For instance, inhibitory immune checkpoint protein PD-L1 and LAG-3 were negatively correlated with *Lactobacillus* abundance in CVM, while TLR2 was in positive relationship with *Lactobacillus* abundance. Conversely, PD-L1 and LAG-3 positively correlated to dysbiosis-associated *Gardnerella*, *Sneathia*, *Atopobium* and *Prevotella*. At species level, *L. crispatus* and *L. jensenii* were in negative relationship with PD-L1, while *L. gasseri* was negatively associated with LAG-3. In addition, a 12-month observational study applied the combination of 16S rDNA amplicon sequencing, metagenome, transcriptional profiling and immunological profiling to demonstrate the critical role of cervicovaginal bacteria in modulating cervicovaginal immune responses and the host susceptibility to HIV (23).

## Application of CVM in Promoting HPV Clearance

**Figure 3:**
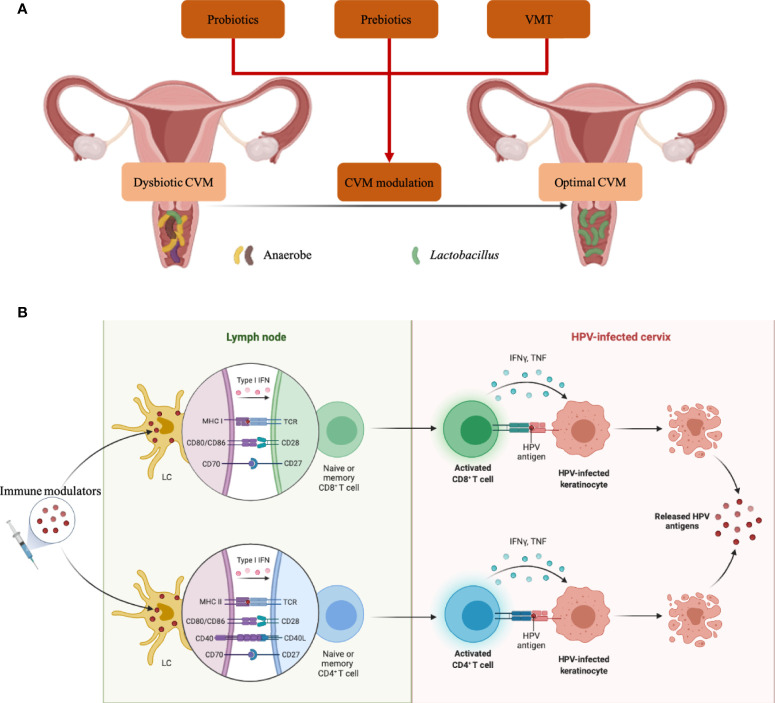
CVM-modulated and -derived products in eliminating HPV infection. **(A)** Strategies to modulate dysbiotic CVM were characterized by anaerobe overgrowth. **(B)** Molecules produced by specific microbial strains in CVM can activate LCs in the cervical epithelium, then promote T cell priming and eliminate HPV-infected cells. Icons in BioRender.com were utilized to prepare this figure.

Given the critical roles of CVM in modulating cervical immune responses, it is promising to promote HPV clearance by re-constructing CVM (**Figure 3A**). Taken vaginal probiotics *L. crispatus* strain CTV-05 for example, a randomized placebo-controlled clinical trial showed that the vaginal colonization with CTV-05 following 28-day treatment inhibited BV-associated *Atopobium* growth (89, 90). Another trial involving 100 participants assessed the efficacy of CTV-05 on preventing urinary tract infection (UTI), indicating the reduction of recurrent UTI when compared to placebo treatment (91). Disrupting biofilm of anaerobes is also an alternative therapy against vaginal dysbiosis, and Marrozzo J. M. et al. found 50-59% clinical cure rate of BV in 106 participants 9-12 days after treatment (92).

In addition, several *in vitro* and *in vivo* studies suggest the promise of prebiotics, which are indigestible carbohydrates, in promoting the growth of probiotics or beneficial commensals in the vagina (**Figure 3A**) (93–95). For example, fructo-oligosaccharide (FOS) and gluco-oligosaccharide (GOS) benefited the growth of *L. crispatus*, *L. jensenii* and *L. vaginalis in vitro*, while pathogen *C. albicans*, *Escherichia coli* and *G. vaginalis* could not utilize FOS/GOS as energy sources for growth (93). Significant reduction of Nugent scores was also identified in BV patients receiving intravaginal GOS gel immediately following metronidazole treatment (94). Additionally, glucomannan hydrolysates (GMH) also held the potential to promote *Lactobacillus* spp. colonization, conferring health to the host in *C. albicans-*infected women (95). To re-establish the CVM completely, two studies in 2019 conducted vaginal microbiota transplantation (VMT) (**Figure 3A**) (96, 97). A total of 5 women with antibiotic-unresponsive and recurrent BV were included in one study, and 4 out of 5 participants had restoration of *Lactobacillus*-dominant CVM and long-term remission without any adverse effect at the follow-up of 5-21 months. The other study involving 20 women explained and implemented a screening approach for universal VMT donors.

Besides to CVM-targeted interventions, CVM-derived products hold the promise as immune modulators, such as adjuvants of therapeutic vaccines (**Figure 3B**). Jie Song and co-workers demonstrated that PGN produced by a vaginal *L. crispatus* strain enhanced the expression of cell-membrane TLR2 and TLR6 to activate LCs (25), which play a pivotal role in capturing and presenting HPV antigens. The products of specific bacterial components have the potential to be effective adjuvants as a series of clinical trials demonstrated enhanced efficacy of therapeutic vaccines adjuvanted with TLR agonists which could be served by bacterial products (98–101). Furthermore, bacterial vectors are increasingly explored as alternative live vectors due to their potential as “natural” adjuvants, which attributed to the wide range of pathogen-associated molecular pattern molecules and damage-associated molecular pattern molecules (102–106). Additionally, candin produced by common vaginal fungal pathobiont *Candida* could be utilized as adjuvant for therapeutic vaccine, which partly explain the protection of vaginal *Candida* against HPV infection (44, 83, 84).

## Conclusion

CVM appears to play a crucial role in HPV acquisition and persistence as well as subsequent development of squamous intraepithelial lesion. Cross-sectional nature of most studies makes it difficult to derive a causal link between CVM and HPV infection or clearance. In addition, many prior reports described CVM in relatively small cohorts, which analysis results could be compounded by various factors, such as smoking and sex activities. Prospective cohort study will be needed in the future to prove that CVM could prevent HPV infection and promote HPV clearance. This information will determine the promise of CVM interventions as novel therapies, with the advantage of low-cost feasibility in developing countries. Nevertheless, it is imperative to find the most protective strains before developing CVM-targeted probiotics or prebiotics, for which the efficacy can be impacted by pre-existed CVM. For example, *L. crispatus*-dominated CVM confers high colonization resistance to other microbes and even probiotic *L. crispatus* strain, while pre-colonization of the vagina with endogenous *L. iners* allows growth of anaerobes. Therefore, CVM structure should be taken into consideration when it comes to assess the efficacy of specific probiotics and prebiotics. However, 16S rDNA amplicon sequencing that most studies applied has limitations in conducting strain-level analysis and microbe-microbe/host interactions of CVM, necessitating the utilization of multi-omics in analyzing “key microbial strains”. Then mechanistic studies of these strains should be conducted to further the utilization of “key microbial strains” as immune modulators in prevention and clearance of HPV infection. Given the importance of cervical epithelial LCs in presenting HPV antigens to induce HPV-specific CMI, it will be an effective mediator of therapeutic vaccine immunity. As discussed above, specific microbial strains in CVM hold the potential to activate HPV-suppressed LCs, suggesting the promise of microbial products as robust activator of immunity against HPV or adjuvants in therapeutic vaccines. In the future, the combination of culture-independent and -dependent techniques should be applied to screen promising microbial strains and products which functions can be assessed in cell lines or animal models. Lastly, though VMT can modify the whole cervicovaginal microenvironment, randomized, placebo-controlled studies for large cohorts are required to determine the clinical efficacy as well as long-term benefits.

## Author Contributions

WD and RW made substantial contributions to the design and writing of this manuscript. HD and SL contributed to the discussion and conception of the work. All authors contributed to the article and approved the submitted version.

## Funding

This work was supported by Shenzhen High-level Hospital Construction Fund (YBH2019-260), Shenzhen Key Medical Discipline Construction Fund (No. SZXK027) and Sanming Project of Medicine in Shenzhen (No. SZSM202011016).

## Conflict of Interest

The authors declare that the research was conducted in the absence of any commercial or financial relationships that could be construed as a potential conflict of interest.

## Publisher’s Note

All claims expressed in this article are solely those of the authors and do not necessarily represent those of their affiliated organizations, or those of the publisher, the editors and the reviewers. Any product that may be evaluated in this article, or claim that may be made by its manufacturer, is not guaranteed or endorsed by the publisher.
